# Malignant Thyroid-type Papillary Neoplasm in Struma Ovarii: A Case Report

**DOI:** 10.7759/cureus.6450

**Published:** 2019-12-23

**Authors:** Syed Adeel Hassan, Ali Akhtar, Noor Ul Falah, Fahad N Sheikh

**Affiliations:** 1 Internal Medicine, Dow University of Health Sciences, Karachi, PAK; 2 Internal Medicine, Shaukat Khanum Memorial Cancer Hospital and Research Centre, Lahore, PAK; 3 Pathology, Shaukat Khanum Memorial Cancer Hospital and Research Centre, Lahore, PAK; 4 Internal Medicine, Sahiwal Medical College, Sahiwal, PAK

**Keywords:** papillary thyroid carcinoma, struma ovarii, thyroid gland, ovarian tumor, surgical debulking, psammoma body, dystrophic calcification, orphan annie eye nuclei, ca-125, stained glass appearance

## Abstract

Papillary thyroid carcinoma (PTC) arising in a coexistent struma ovarii (SO) is a rare malignancy. It manifests with abdominal symptoms such as palpable mass, pain, distension, and possibly ascites. It is usually diagnosed postoperatively, and its histopathological diagnostic criteria remain identical to that of papillary carcinoma of the thyroid gland. Due to the relative rarity of the disease, definitive guidelines for its overall management are still undefined. We present a case of a 51-year old female with suspicion of a left ovarian tumor due to her presenting symptoms: raised serum CA-125 levels and abnormal abdominopelvic CT scan findings. She underwent complete surgical debulking of the mass (total abdominal hysterectomy (TAH), bilateral salpingo-oophorectomy (BSO), omentectomy, appendectomy, and pelvic lymphadenectomy). The mass was postoperatively diagnosed by histopathology as PTC in SO (stage IA). Furthermore, our patient did not receive any adjuvant treatment. The patient has been disease-free for 24 months post-surgery and is scheduled for regular biannual follow-ups.

## Introduction

Struma ovarii (SO) is defined as the presence of a single predominant type of mature thyroid tissue in a coexisting ovarian tumor. To date, only about 200 cases have been reported in the literature. It presents more frequently between the ages of 40 and 60 [1]. The majority of SO are benign and belong to the category of mature teratomas, which account for 2-5% of overall benign mature teratomas [2,3]. Rarely, a malignant type of tissue may arise in the background of SO, thereby transforming it into a thyroid-type carcinoma. In such malignant cases, the most common histological subtypes, in the order of decreasing prevalence, include papillary thyroid carcinoma (PTC), follicular thyroid carcinoma, and highly differentiated follicular carcinoma (HDFCO) [3]. Malignant struma ovarii (MSO) are usually asymptomatic. However, thyrotoxicosis is reported to be present in 5-8% of the affected patients [4]. Our case discussion pertains to the rare variant of MSO harboring a focus of the classical or conventional type of PTC. Due to its rarity, sufficient data are not available regarding an approach to diagnosis, treatment, and prognosis. Therefore, our report discusses its clinicopathological features, diagnostic criteria, and management strategies.

## Case presentation

A 51-year-old female (gravida 6, para 6) presented to our walk-in clinic with a four-month history of progressive lower abdominal pain and distention in the lower left quadrant. She complained of indigestion and had had significant weight loss in the last two months. Her past medical history revealed she had been operated for cholelithiasis 14 years ago. The patient's family history was insignificant for other comorbidities and cancer. Physical examination was consistent with a palpable abdominopelvic mass (8-10 weeks in size) on the lower left side.

**Figure 1:**
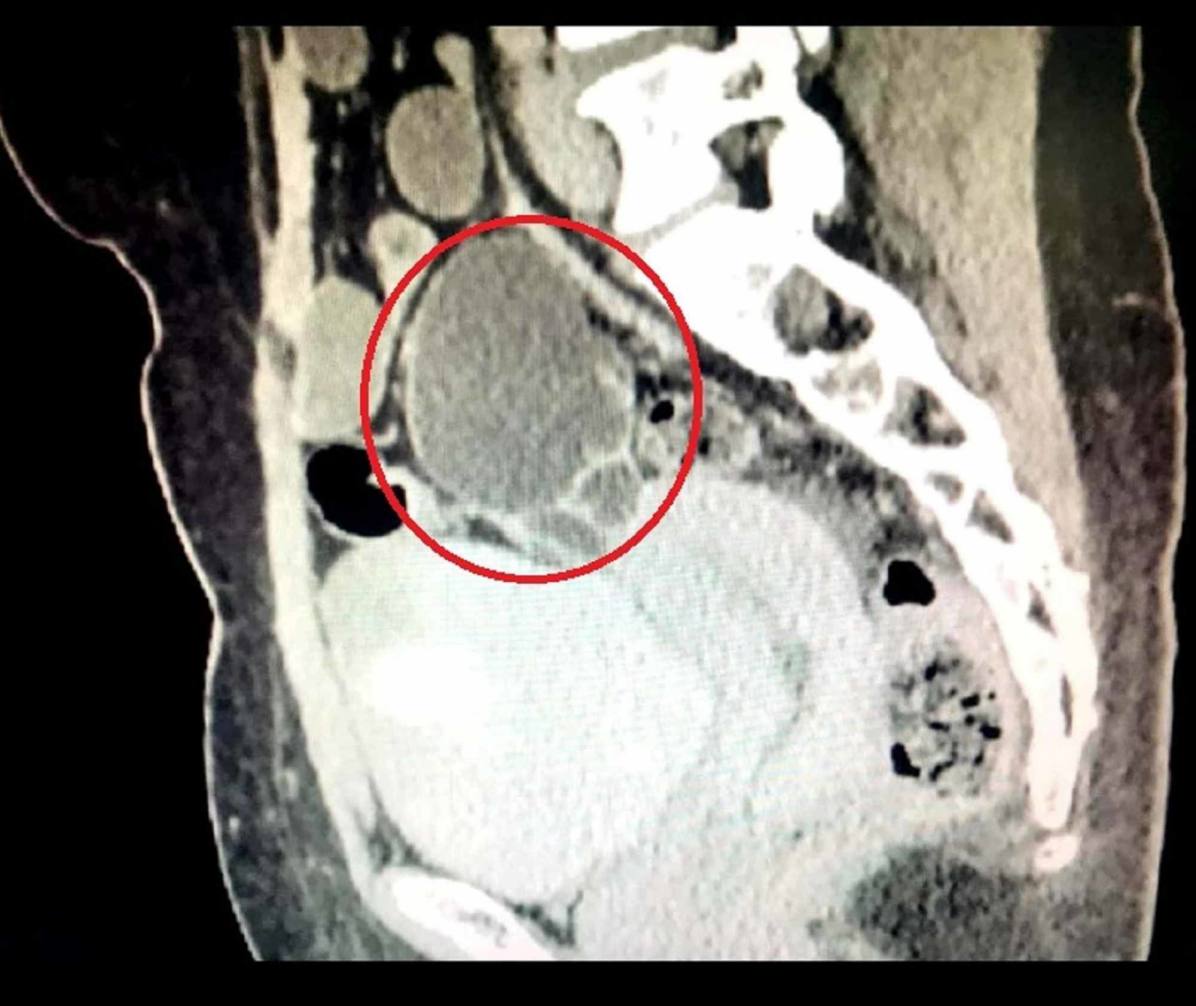
Sagittal CT scan of the abdomen/pelvis Sagittal CT depicting a large left adnexal mass (red circle)

**Figure 2:**
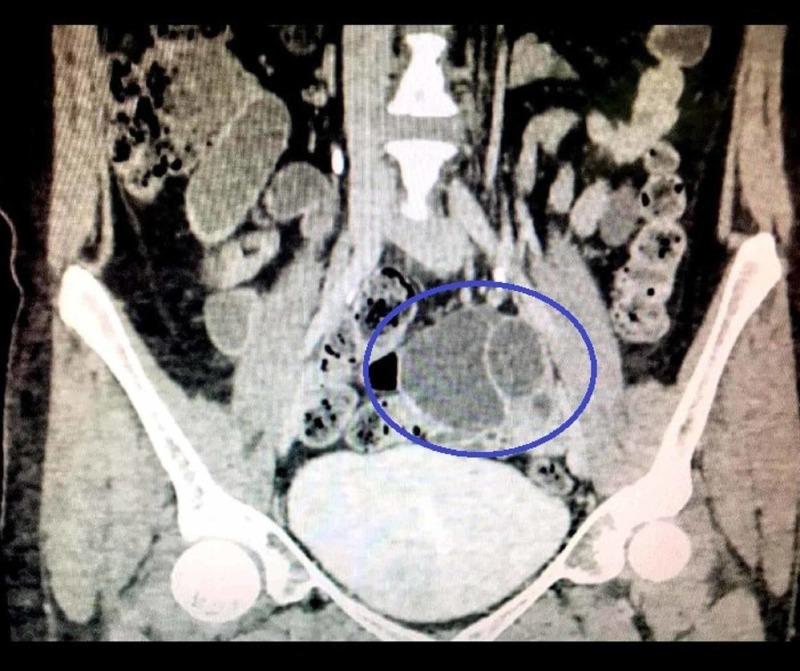
Coronal CT scan of the abdomen/pelvis A left multiloculated adnexal mass (blue circle), with solid and cystic components, is appreciated. The differential signal intensity between locules imparts a stained glass appearance.

A CT scan of the abdomen and pelvis yielded a large left adnexal mass measuring approximately 8.1 x 6.7 cm (Figure 1). The architecture of the mass depicted compositions of both solid and cystic components (Figure 2). The uterus and the right adnexa showed no signs of disease involvement and therefore were deemed to be normal. However, small-to-moderate volume ascitic fluid with peritoneal stranding was noted. No pelvic invasion, lymphadenopathy, or secondary implants were identified. Digital chest X-ray and CT of the chest to rule out systemic spread showed clear lung fields and no pleural involvement. Our patient also tested positive for serum CA-125, whose levels were noted at 413 units/mL (normal range: 0-35 units/mL). The results of other diagnostic tests such as thyroid profile, serum LDH, alpha-fetoprotein, and beta-HCG were within normal limits. Therefore, based on imaging results and serum CA-125 levels, the initial diagnosis of the primary ovarian tumor was made. Subsequently, our patient underwent total abdominal hysterectomy (TAH) with bilateral salpingo-oophorectomy (BSO). Omentectomy, appendectomy, pelvic lymphadenectomy, and peritoneal biopsy were also performed. On gross examination of the surgically resected specimen, a multiloculated solid and cystic left ovarian mass was noted. Uterus, right ovary, and bilateral fallopian tubes showed no gross signs of disease involvement. Adhesions noted between the small bowel and urinary bladder wall were removed via adhesiolysis. The postoperative recovery was uneventful and satisfactory.

**Figure 3:**
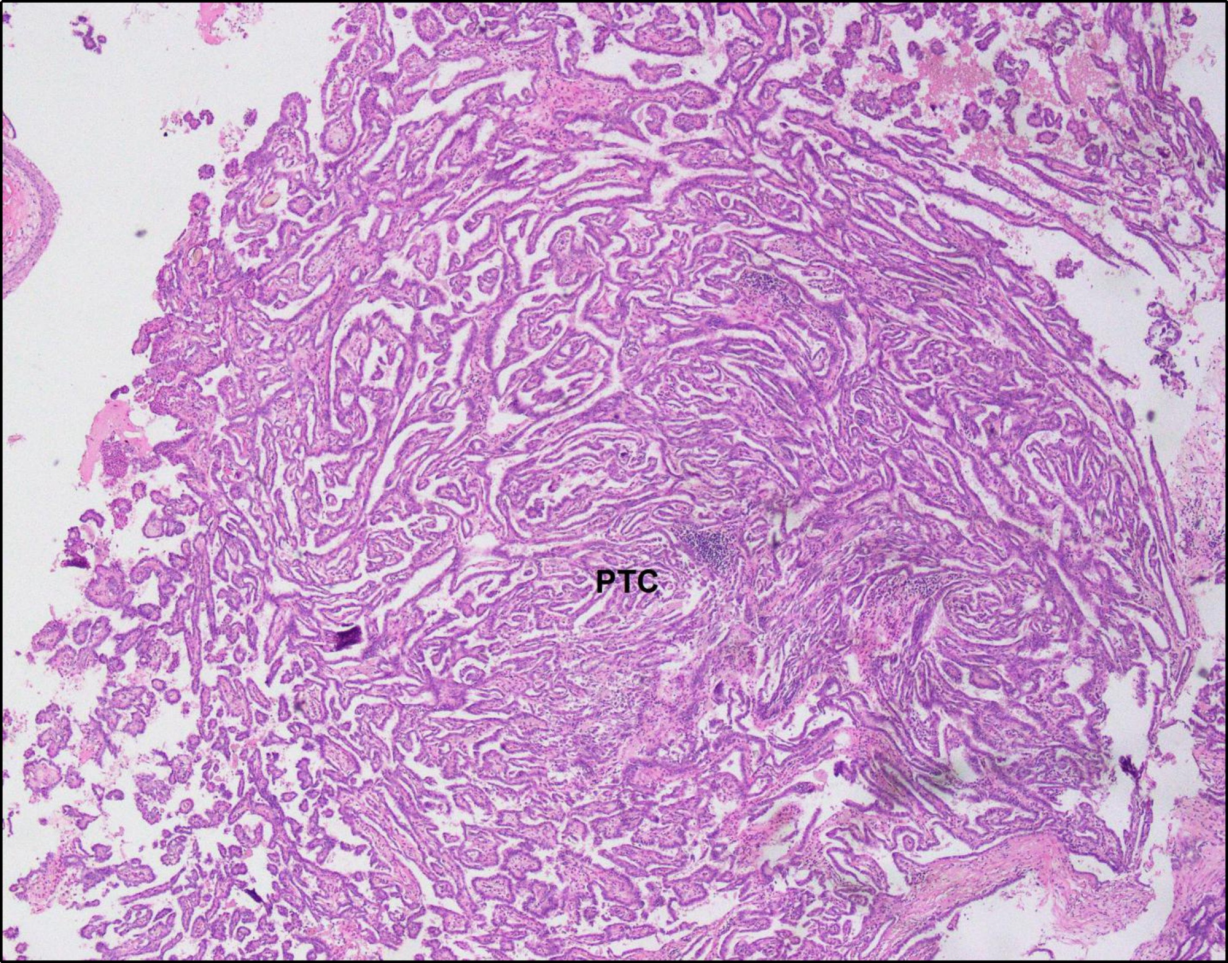
Low-resolution image depicting a focus of PTC in a struma ovarii PTC: papillary thyroid carcinoma Multiple complex and branched papillae with central fibrovascular cores are seen, with presumably dystrophic calcification (psammoma body) in one papilla. The identification of psammoma bodies in a thyroid-type papillary neoplasm is highly supportive of malignancy. However, they are better visualized on a higher resolution

**Figure 4:**
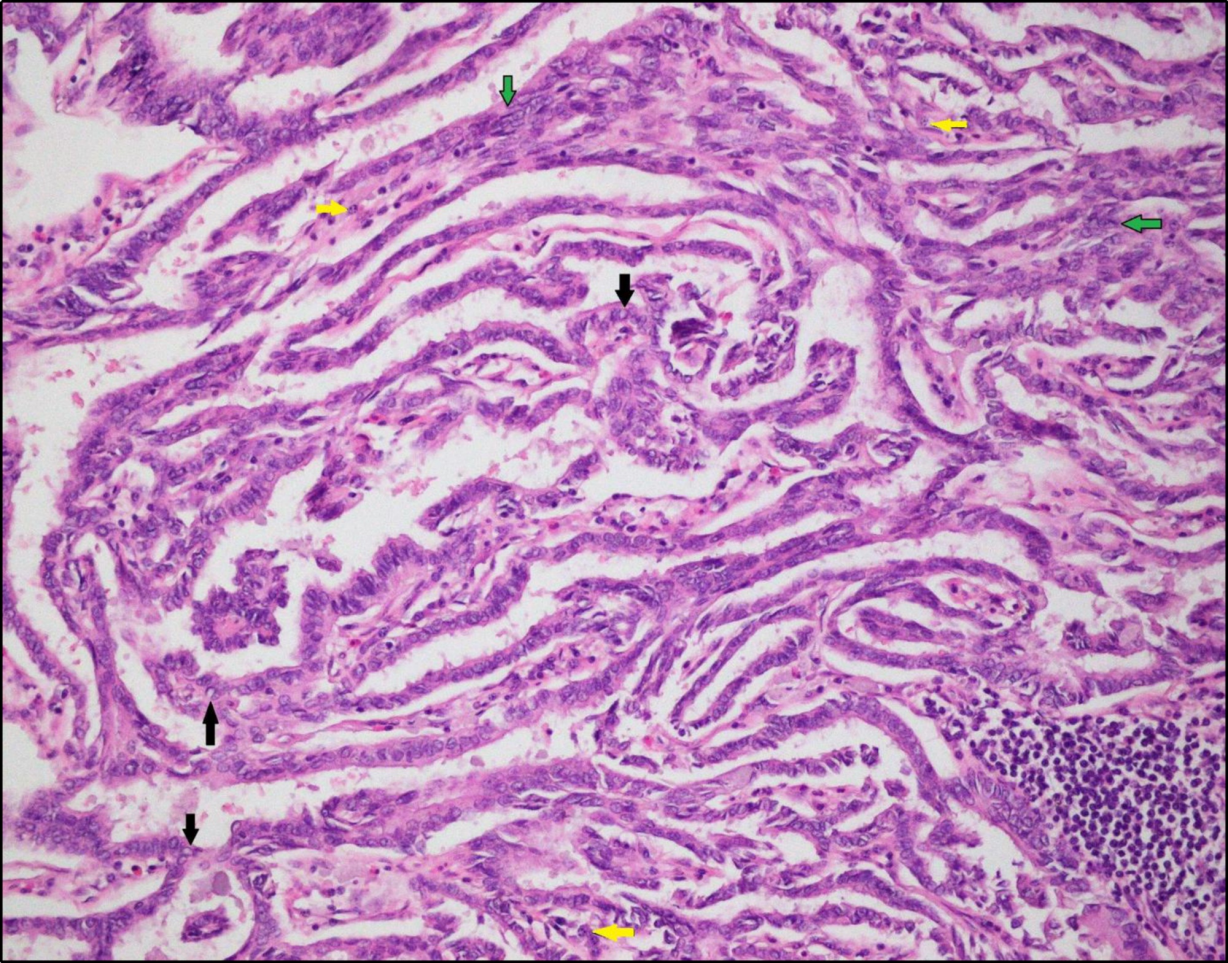
High-resolution image depicting the papillary architecture of the tumor The papillary architecture of the tumor is evident with stromal tissue in the fibrovascular cores. Papillae are lined by cuboidal to low columnar neoplastic cells with characteristic ‘Orphan-Annie eye’ nuclei, having clear ground-glass chromatin (black arrow). Nuclear overcrowding is evident (green arrow). Some nuclei show coffee-bean appearance, with longitudinal grooves due to nuclear membrane infoldings (yellow arrow)

**Figure 5:**
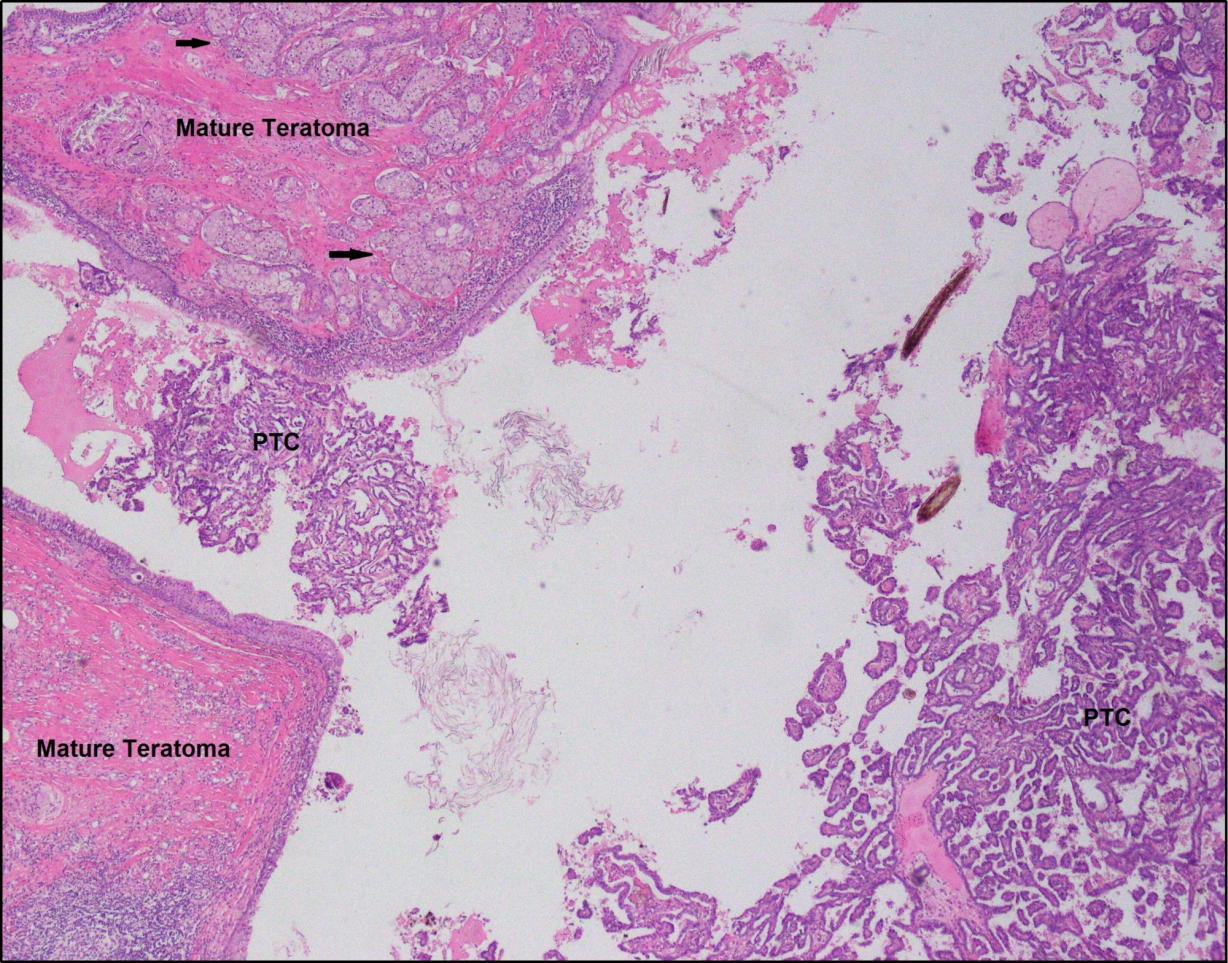
Low-resolution image showing mature teratoma on the left side of the image adjacent to the focus of papillary thyroid carcinoma arising in a struma ovarii PTC: papillary thyroid carcinoma The major bulk of the PTC can be seen on the right side. The benign teratomatous tissue shows dense stroma with multiple, relatively well-circumscribed sebaceous glands (black arrows) and overlying stratified epithelium. Benign-looking thyroid follicular tissue with eosinophilic colloid and flattened epithelial lining can also be seen at the top right corner

Histopathological studies of the left ovarian mass revealed a focus of classical PTC measuring 2 cm in the background of a SO (Figures 3-5). The focus of carcinoma lacked features of surface involvement or capsular rupture. Right adnexa, left fallopian tube, endometrium, appendix, omentum, pelvic lymph nodes, and peritoneum showed no signs of malignant involvement. Therefore, the tumor was limited to one ovary with its capsule intact and showed no signs of involvement on the ovarian surface. As per the International Federation of Gynecology and Obstetrics (FIGO) system for ovarian cancer staging, our specimen was considered IA (pT1aN0M0). Immunohistochemistry was not pursued owing to the histological confirmation of the tumor. Due to a lack of metastasis and localization of the tumor in the left ovary, it was decided that the patient should not be given any form of adjuvant therapy. Postoperatively, the patient was surveilled for thyroid carcinoma with the help of thyroid panel tests and thyroid gland ultrasonography (done twice). The results of the surveillance were normal.

Our patient was scheduled for biannual follow-ups and is now disease-free two years post-resection. Postoperative serial measurements of thyroglobulin, anti-thyroglobulin antibody, serum CA-125 levels and contrast CT imaging of chest, abdomen, and pelvis have been conducted. CT has not shown any residual or recurrent disease so far. The patient is euthyroid with normal postoperative thyroglobulin and anti-thyroglobulin antibody levels. Serum CA-125 levels have shown an initial decreasing trend post-surgery. However, during the second follow-up visit, a raised serum CA-125 level was noted, which regressed to baseline during the subsequent follow-ups. The preoperative and postoperative progression patterns of serum CA-125 levels are noted below in Table 1. 

**Table 1:** Postoperative follow-up of patient’s serum CA-125 levels CA 125: cancer antigen 125; normal serum CA 125: <35 U/mL

Patient’s stage	Serum CA-125 level (U/mL)
Preoperative	413
One month post-surgery	26.7
First follow-up	18.3
Second follow-up	38.3
Third follow-up	18
Fourth follow-up	14.7

## Discussion

Germ cell tumors of the ovary are known to arise from any form of pathogenic insult to the normal ovarian germ cell lineage. Such disruption in physiological growth can occur during any stage of ovarian germ cell maturation ultimately leading to neoplastic transformation [1]. Table 2 lists the common genetic mutations leading to PTC in SO. The majority of ovarian germ cell tumors are benign, of whom mature teratomas make up 15-20% of the cases [2]. In comparison to its benign counterpart, the incidence of malignant ovarian germ cell tumors is small [1,3]. Teratoma is a subtype of germ cell tumor that arises from one or more of the three germ cell layers. They can present as benign cystic lesions to overt malignancies [4]. Malignant counterparts of teratomas are termed immature teratomas. Less than 3% of ovarian teratomas are malignant, and they usually occur in postmenopausal females [5]. An estimated 15% of teratomas are known to contain thyroid tissue [2]. SO is a monodermal ovarian teratoma, whose architecture is composed of more than 50% of thyroid tissue [3,6]. It contributes to approximately 2-3% of all ovarian tumors and 2-5% of all ovarian teratomas [1-4]. The incidence of SO is higher in older adults, although it has also been reported in younger women [7]. It usually has an asymptomatic presentation. However, it may also produce non-specific symptoms of a pelvic mass such as abdominal pain, bloating, or abnormal menstrual cycles [6-8]. The majority of SO cases are benign, with only 5-10% cases being malignant [4]. Therefore, a malignancy arising in a SO is a rare event [4]. The average age of females at the diagnosis of MSO is 43 years [2]. Histologically, papillary and follicular carcinoma of the thyroid is the two most common subtypes of malignancies arising in a SO. Studies including those of Szczepanek-Parulska E, et al., Zhu Y, et al., and Leite, I et al. have shown that PTC is the more common of the two presenting histological subtypes [1,2,9].

**Table 2:** Genetic mutations in struma ovarii leading to papillary thyroid carcinoma  formation PTC: papillary thyroid carcinoma BRAF gene: a proto-oncogene that encodes the B-RAF protein V600E: missense mutation of the BRAF gene, where valine (V) is substituted by glutamic acid (E) at amino acid 600 G469A: missense mutation of the BRAF gene within exon 11 K601E: mutation of the BRAF gene, where amino acid substitution occurs at position 601 KRAS gene: a proto-oncogene that encodes the K-RAS protein Q61R: mutation of the KRAS gene, where glutamine (Q) is substituted by arginine (R) at position 61 NRAS gene: a proto-oncogene that encodes the N-RAS protein PTEN gene: phosphate and tension homolog gene which encodes an enzyme with tumor suppressor activity RET/PTC: rearrangement of the RET gene

Gene mutation	Study (author, publication year, citation)
BRAF (V600E) present in two-thirds of cases	Zhu Y et al. (2016) [2], Zhang T et al. (2018) [6]
BRAF (G469A), KRAS (Q61R)	Tzelepis EG et al. (2019) [4}
BRAF (K601E), NRAS, PTEN, RET/PTC	Zhang T et al. (2016} [2], Tzelepis EG et al. (2019) [4]

Due to the rarity of MSO and its available literature data, no formal standard criteria have been established regarding its diagnosis, management, and follow-up [1,2]. Therefore, the diagnosis of PTC in a SO requires a detailed history followed by a comprehensive clinical examination. Furthermore, imaging studies can be supported with histopathological analysis, immunohistochemistry, and serum tumor markers. Most tumors remain clinically indolent without producing any specific symptoms [1]. Commonly, a palpable pelvic mass, pelvic pain, or discomfort are the presenting symptoms [1]. Some patients may also develop abdominal distension due to ascites [3,6]. Relevant to our case, our patient presented with a palpable pelvic mass and also demonstrated a moderate volume of ascitic fluid on CT. Therefore, the aforementioned clinical signs are consistent with our findings. Thyrotoxicosis is uncommon and seen only in 5-8% of the affected patients having functional MSO [4]. Therefore, on average, 92% of the patients remain euthyroid, a finding that is further consistent with our case [1].

Confirmation of PTC in a SO is usually postoperative because it requires a histopathological analysis for its exact identification [6]. According to Szczepanek-Parulska E, et al., the criteria used for its identification are the same as that used for the histological diagnosis of primary papillary carcinoma of the thyroid gland [1]. These histological criteria include, but are not limited to, the presence of papillary architecture of the tumor with stromal tissue in the fibrovascular cores, papillae lined by cuboidal to low columnar neoplastic cells, characteristic ‘Orphan-Annie eye’ nuclei having clear ground-glass chromatin, nuclear overcrowding, and longitudinal grooves due to nuclear membrane infoldings. These criteria are fulfilled and depicted on our patient's specimen biopsy. It is also noteworthy that radiological findings are non-specific [1].

Patients can also have high levels of CA-125, a finding also consistent with our patient (413 U/ml preoperatively) [4]. However, according to Szczepanek-Parulska E, et al. and Zhang T, et al., raised CA-125 levels have low significance because they can be elevated in both benign and malignant carcinomas of the ovary, contributing little to the diagnosis [1,6]. Factors such as thyroglobulin, thyroid transcription factor 1, cytokeratin 7, cytokeratin 19, neuron-specific enolase, and p63 can be positive on immunohistochemistry, aiding in diagnosis [1,3]. Anagnostou E, et al. and Leite I, et al. described PTC in SO to have a low metastatic potential [3,9]. Coexisting primary PTC is also a rare possibility [10]. Therefore, imaging techniques such as high-resolution ultrasonography, CT, and MRI should be used to scan the thyroid gland to exclude its carcinoma and thereby ovarian metastasis [2].

No definitive management guidelines regarding PTC in SO have been proposed so far. The decision about the surgical treatment of MSO depends mainly on the need to preserve the fertility of the patient and the extent of the tumor. A unilateral salpingo-oophorectomy is recommended if the patient wants to preserve fertility, the tumor is confined to the ovary, and if there is no evidence of thyroid nodules [2,4]. When there is no such need to preserve fertility and/or in cases where the tumor has metastasized, aggressive management is advised with TAH/BSO, lymph-node dissection, and omentectomy [2,4,10]. The guidelines for postoperative adjuvant treatment are not clearly defined. However, some suggest adjuvant therapy in advanced cases, comprising external radiotherapy, chemotherapy, and thyroid suppression therapy [2]. Near-total thyroidectomy followed by radioactive iodine ablation (RIA) is not always applied but is recommended in patients with metastatic disease and/or a higher risk of recurrence [2-4,6]. Thyroidectomy not only potentiates the subsequent RAI but also helps in measuring thyroglobulin levels as a marker for metastases, residual mass, or recurrence [4,6-8]. Thyroidectomy followed by RAI in cases of surgical resection of MSO gives variable tumor recurrence rates [4]. Some studies suggest the use of thyroxine to suppress thyroid-stimulating hormone (TSH) post-surgically in a patient with non-metastasized tumor after its surgical resection [3]. However, this has not been documented persistently enough in literature to determine its impact on recurrence rates [2]. Therefore, this form of therapy was not carried out in our case. In summary, the decision regarding total thyroidectomy, radioactive iodine therapy, and thyroxine suppression therapy should depend on the individual cases and their clinical parameters.

Studies have shown a favorable postoperative prognosis for non-metastasized MSO. A study conducted by Goffredo P et al., which delved into 68 patients, indicated survival rates of 96.7%, 94.3%, and 84.9% at 5, 10, and 20 years respectively [10]. Furthermore, a study conducted by Robboy et al., which looked at 88 cases, showed a survival rate of 89% at 10 years and 84% at 25 years for all the patients [11]. The complete surgical removal of a hormonally inactive, stage IA tumor shows a good prognosis. Long-term follow-up care is essential due to its probability of recurrence. The average duration of initial recurrence is reported at 4 years [6]. A tumor of size 2 cm or less carries a low chance of recurrence. Imaging is important to rule out disease recurrence in cases of varying CA-125 levels.

We strongly recommend presenting patients with a plan to adhere to a regular biannual or triannual follow-up with abdominopelvic CT imaging with contrast and serum CA-125 monitoring. Postoperative thyroid carcinoma surveillance is further essential to exclude ovarian metastasis as a possibility, and this can be achieved with serial measurements of thyroglobulin and antithyroglobulin antibody levels. Follow-up surveillance for possible metastasis should also include contrast CT imaging of the chest.

## Conclusions

We reported a rare case of a 51-year-old female, who presented with a four-month history of progressive lower abdominal pain and a mass (8-10 weeks in size) in the left lower quadrant. With the aid of CT imaging and histopathological studies, the diagnosis of MSO was confirmed. Our case report discusses the etiology, clinical presentation, and management strategies of MSO. Due to the chances of metastasis and recurrence, we also lay stress on the importance of implementing postoperative follow-up surveillance. Furthermore, we also reiterate that, given the rarity of MSO, no formal standard criteria have been established. This makes the management of such cases a real clinical challenge. 

## Appendices

Abbreviations:

PTC: papillary thyroid carcinoma

SO: struma ovarii

MSO: malignant struma ovarii

FIGO: International Federation of Gynecology and Obstetrics

TAH: total abdominal hysterectomy

BSO: bilateral salpingo-oophorectomy

CT: computerized tomography

MRI: magnetic resonance imaging

## References

[1] Szczepanek-Parulska E, Pioch A, Cyranska-Chyrek E (2019). The role of immunohistochemical examination in diagnosis of papillary thyroid cancer in struma ovarii. Folia Histochem Cytobiol.

[2] Zhu Y, Wang C, Zhang GN, Shi Y, Xu SQ, Jia SJ, He R (2019). Papillary thyroid cancer located in malignant struma ovarii with omentum metastasis: a case report and review of the literature. World J Surg Oncol.

[3] Anagnostou E, Polymeris A, Morphopoulos G, Travlos A, Sarantopoulou V, Papaspyrou I (2016). An unusual case of malignant struma ovarii causing thyrotoxicosis. Eur Thyroid J.

[4] Tzelepis EG, Barengolts E, Garzon S, Shulan J, Eisenberg Y (2019). Unusual case of malignant struma ovarii and cervical thyroid cancer preceded by ovarian teratoma: case report and review of the literature. Case Rep Endocrinol.

[5] Sinha A, Ewies AA (2019). Ovarian mature cystic teratoma: challenges of surgical management. Obstet Gynecol Int.

[6] Zhang T, Chen P, Gao Y (2019). Struma ovarii: a mini review. Int J Clin Exp Med.

[7] Iranparvar Alamdari M, Habibzadeh A, Pakrouy H, Chaichi P, Sheidaei S (2019). An unusual presentation of a papillary thyroid carcinoma in the struma ovarii in a 10 year-old girl: a case report. Int J Surg Case Rep.

[8] Middelbeek RJW, O'Neill BT, Nishino M, Pallotta JA (2019). Concurrent intrathyroidal thyroid cancer and thyroid cancer in struma ovarii: a case report and literature review. J Endocr Soc.

[9] Leite I, Cunha TM, Figueiredo JP, Félix A (2019). Papillary carcinoma arising in struma ovarii versus ovarian metastasis from primary thyroid carcinoma: a case report and review of the literature. J Radiol Case Rep.

[10] Goffredo P, Sawka AM, Pura J, Adam MA, Roman SA, Sosa JA (2019). Malignant struma ovarii: a population-level analysis of a large series of 68 patients. Thyroid.

[11] Robboy SJ, Shaco-Levy R, Peng RY (2009). Malignant struma ovarii: an analysis of 88 cases, including 27 with extraovarian spread. Int J Gynecol Pathol.

